# mGlu5 Receptors and Relapse to Cocaine-Seeking: The Role of Receptor Trafficking in Postrelapse Extinction Learning Deficits

**DOI:** 10.1155/2016/9312508

**Published:** 2016-01-10

**Authors:** Lori A. Knackstedt, Marek Schwendt

**Affiliations:** Psychology Department, University of Florida, Gainesville, FL 32611, USA

## Abstract

We have previously demonstrated that MTEP, an allosteric antagonist of mGlu5, infused into the nucleus accumbens attenuates relapse after abstinence from cocaine self-administration. MTEP infused into the dorsolateral striatum (dlSTR) does not alter relapse but has long-lasting effects on subsequent extinction learning. Here we tested whether systemic MTEP would prevent relapse after abstinence or alter extinction learning. We also investigated the mechanism of action by which intra-dlSTR MTEP on test day alters extinction on subsequent days. Animals self-administered cocaine for 12 days followed by abstinence for 20-21 days. MTEP (0.5–5 mg/kg IP) was administered prior to placement into the operant chamber for a context-primed relapse test. A separate group of animals received intra-dlSTR MTEP prior to the relapse test and were sacrificed day later. Systemic administration of MTEP attenuated abstinent-relapse without significantly affecting extinction learning. Surface biotinylation analysis of protein expression in the dlSTR revealed that, in cocaine animals, intra-dlSTR MTEP administration decreased mGlu5 surface expression and prevented changes in Arc and GluA1/GluA2 observed in their vehicle counterparts. Thus, blockade of mGlu5 receptors may be utilized in future treatment strategies for relapse prevention in humans, although the effects of chronic blockade on extinction learning should be further evaluated.

## 1. Introduction

Cocaine addiction is a chronic relapsing disorder characterized by loss of control over drug-seeking. A major challenge in the successful treatment of cocaine addiction is the risk of relapse, which remains high even after months or years of abstinence [1, 2]. Animal models of relapse are essential for elucidating the neurobiological processes that underlie relapse behavior and for assessing the effectiveness of pharmacotherapies aiming to prevent cocaine relapse.

The extinction-reinstatement paradigm is one such animal model. Drug self-administration occurs in an operant chamber and is followed by extinction training in which operant responses that previously yielded drug delivery are no longer reinforced and the operant behavior declines. The drug-seeking response can be reinstated by exposure to stimuli known to cause relapse in humans, including stress [3], discrete cues previously paired with drug delivery [4], and/or the drug itself [5]. In the abstinent-relapse model, animals are not subjected to extinction training following self-administration but instead experience abstinence in the home cage. Animals are then reexposed to the drug taking environment for a context-primed relapse test. During this time drug and discrete cues are not delivered [for review see [6]]. Because extinction of the operant response does not occur in this model, the response cannot be “reinstated.” Therefore, this test is referred to as an abstinent-relapse or context-primed relapse test [7, 8].

Pharmacological inhibition of the dorsolateral striatum (dlSTR) attenuates relapse following abstinence [8] but has no effect on cue-primed reinstatement after extinction training [9]. The nucleus accumbens (NAc) has been reported to be unnecessary for abstinent-relapse when animals are trained to self-administer cocaine in the absence of discrete cues [10]. However, when the drug-seeking response results in drug delivery combined with discrete drug-associated cues (light + tone), the NAc mediates abstinent-relapse [7]. The NAc is also essential for context-primed renewal of drug-seeking when extinction occurs in a context different from the self-administration context [11].

Glutamate dysregulation in the NAc core has been identified as a primary driver of drug-seeking behavior after extinction training (for review see [12]). We previously demonstrated that the infusion of MTEP, a negative allosteric modulator (NAM) of metabotropic glutamate receptor subtype 5 (mGlu5 or mGluR5), into the NAc attenuates relapse following abstinence without extinction. In the same publication we also demonstrated that intra-dlSTR MTEP given only on the relapse test day did not block relapse but produced deficits in extinction learning that persisted for four days [7]. We have also shown that cocaine self-administration and abstinence result in decreased mGlu5 surface levels in the dlSTR [7]. As mGlu5 represents an important target for preventing drug-seeking, here we investigated the ability of a single administration of systemic MTEP to both attenuate relapse and alter future extinction learning. We hypothesized that the mechanism by which intra-dlSTR MTEP exerts long-lasting detrimental effects on extinction learning is by decreasing surface expression of mGlu5 within this brain region. To that end, we quantified surface and total protein expression of mGlu5 and its associated scaffolding and signaling protein partners. This assessment was made 24 hr following intra-dlSTR MTEP infusion, at the time at which animals would have experienced the first extinction session after MTEP infusion.

## 2. Materials and Methods

### 2.1. Animals and Drugs

Adult male Sprague Dawley rats (Charles River Laboratories, Raleigh, NC, USA) weighing 275–300 g were single-housed in a temperature and humidity controlled vivarium on a reversed 12-hour light/dark cycle with water available* ad libitum*. Animals were restricted to 20–25 g of standard chow per day. Experiment 1 was performed at the University of Florida and Experiment 2 was performed at the Medical University of South Carolina (MUSC). All animal procedures were approved by the Institutional Animal Care and Use Committees of the University of Florida and MUSC and were performed in accordance with the Guide for the Care and Use of Laboratory Animals. Seventy-four rats were used for this experiment. Experiment 1 used 43 rats, 6 of which were removed from the study due to a loss of catheter patency. Experiment 2 used 34 rats, 3 of which were removed from the study due to a loss of catheter patency or intracranial cannula failure.

Cocaine-HCl was acquired from the NIDA Controlled Substances Program (Research Triangle Institute, NC, USA). Cocaine (4 mg/mL; 0.25 mg/infusion) was dissolved in saline (0.9% sodium chloride). MTEP hydrochloride (0.5, 1.5, and 5 mg/kg, i.p.) was purchased from Abcam Biochemicals (Cambridge, MA, USA) and dissolved in physiological saline (for i.p. administration) or aCSF (artificial cerebrospinal fluid; for intracranial administration) that included 10% Tween-80. MTEP solutions were neutralized to pH 6-7 with 1 N NaOH prior to administration. The concentrations of the drugs used were determined based on the results of previously published studies [7, 14–16].

### 2.2. Catheter and Stereotaxic Surgery

Animals were surgically implanted with jugular vein catheters as described previously [7]. Animals were anesthetized using a mixture of ketamine (87.5 mg/kg, i.p.) and xylazine (5 mg/kg, i.p.). Ketorolac (2 mg/kg, i.p.) was administered pre- and postoperatively for pain. For Experiment 2, following catheter implantation, animals were placed in a stereotaxic frame (Stoelting, Wood Dale, IL, USA) for intracranial guide cannula implantation. Cannulas (20 gauges; Plastics One) were implanted 2 mm above the dlSTR according to the following coordinates: +1.2 mm AP, ±3.4 mm ML, and −3.4 mm DV, relative to bregma according to [13]. Cannulas were secured to the skull with stainless steel skull screws and dental acrylic. Catheters were flushed with 0.2 mL of heparinized saline (100 U/mL) before and after each self-administration session to ensure continued catheter patency. Animals were allowed to recover for 5 days before self-administration procedures were initiated. Catheter patency was verified periodically by intravenous administration of methohexital sodium (10 mg/mL) which produces a temporary and observable loss of muscle tone.

### 2.3. Cocaine Self-Administration, Abstinence, and Relapse Procedures

Animals self-administered cocaine using an FR1 schedule of reinforcement in standard operant chambers (30 × 24 × 30 cm; Med Associates, St. Albans, VT, USA) equipped with two retractable levers. Presses on the active lever resulted in an intravenous infusion of cocaine and a 5-second presentation of auditory (2900 Hz tone) and visual (stimulus light) conditioned cues. Each infusion of cocaine was followed by a 20-second “time-out” period when presses on the active lever produced neither drug nor cue presentation. Presses on the inactive lever were not reinforced but were recorded. Daily sessions (2 hr/day) continued until meeting the criterion of 10 or more infusions for 12 days. Animals then entered the abstinence phase of the experiment where they were weighed daily and removed from their housing room but not placed into the self-administration chamber. Following 20-21 days of abstinence, a context-primed relapse test was conducted during which animals were placed into the operant chambers and levers were extended but presses did not produce drug or cues.

*Experiment 1: MTEP and Context-Primed Relapse*. After 20-21 days of abstinence, animals received a single injection of MTEP (0.5, 1.5, or 5 mg/kg, i.p.) or vehicle (10% Tween-80 in physiological saline) 15 minutes prior to the relapse test. The relapse test also served as day 1 of extinction training as this was the first day that lever presses did not produce drug. Animals were run under extinction conditions for 4 additional days (2 hr/day).

*Experiment 2: Intra-dlSTR MTEP, Context-Primed Relapse, and mGlu5 Surface Expression*. Animals were trained to self-administer cocaine as described above. An additional group of animals was served as “yoked-saline” controls such that they received an infusion of saline when their yoked counterpart self-administered an infusion of cocaine. After 20-21 days of abstinence, both saline and cocaine animals received either a single intra-dlSTR injection of MTEP (5 *μ*g/side) or vehicle (10% Tween-80 in aCSF). Fifteen minutes after infusion, animals were placed in the self-administration chamber for 2 hr relapse test (corresponding to extinction day 1) as described in Experiment 1. Twenty-two hours later, at the time animals would have been placed back into the operant chamber for day 2 extinction test, animals were euthanized by rapid decapitation and brains extracted for surface biotinylation and immunoblotting analysis as described below.

### 2.4. Slice Biotinylation and Immunoblotting

Immediately after the brain extraction, 2 mm thick coronal slices containing the signs of microinjection point of entry into the brain were prepared using the rat brain matrix (ASI instruments, Warren, MI, USA). The dSTR was bilaterally dissected from each slice using a 2 mm micropunch (Harris-Unicore, Ted Pella, Redding, CA, USA) as illustrated in Figure 3(a). Subsequently, 250 *μ*m thick acute slices were prepared from the dSTR tissue (McIlwain Tissue Chopper, Ted Pella, Redding, CA, USA) and the presence of cannula tracks in acute dSTR slices was visually verified. dSTR slices were then subjected to surface biotinylation followed by immunoblotting analysis as described previously [7]. Proteins of interest were analyzed by immunoblotting in total tissue lysates (T), as well as in intracellular (I) and surface (S) fractions prepared by surface biotinylation. Briefly, equal aliquots from each fraction were separated by SDS-PAGE (4–15%) and transferred onto PVDF membrane. Membranes were blocked for 1 h in 5% milk/Tris-buffered saline and probed overnight at 4°C with primary antibodies diluted in 5% milk/Tris-buffered saline with 0.1% Tween 20. The following primary antisera were used: anti-mGlu5 (1 : 5,000), anti-GluA2 (1 : 1000), anti-tyrosine hydroxylase (1 : 20,000; all Millipore, Billerica, MA, USA), ani-GluA1 (1 : 10,000), anti-Homer 1b/c (1 : 10,000), anti-syntaxin-1a (1 : 25,000; all Abcam, Cambridge, MA, USA), anti-Arc (1 : 40,000), anti-Homer 2a/b (1 : 1000; both Synaptic Systems, Göttingen, Germany, USA), anti-GRK2 (1 : 1000), anti-Dynamin II/III (1 : 1000), Ca2+/calmodulin-dependent protein kinase II (CAMKII 1 : 1000; all Santa Cruz Biotechnology, Dallas, TX, USA), and anti-calnexin (1 : 20,000; Enzo Life Sciences, Farmingdale, NY, USA). After incubation with an appropriate HRP-conjugated secondary antiserum (1 : 10–20,000; all from Jackson Immuno Research, West Grove, PA, USA), immunoreactive bands on the membranes were detected by ECL+ chemiluminescence reagents on an X-ray film (GE Healthcare, Piscataway, NJ, USA). Subsequently, the blots were stripped and reprobed with anti-syntaxin-1a or anti-calnexin antibody to normalize for unequal loading and/or transfer of proteins in surface and total fraction, respectively. Membranes were also reprobed with anti-tyrosine hydroxylase antibody to monitor the specificity of biotinylation for surface versus intracellular proteins. The integrated band density of each protein sample was measured using NIH Image J software (http://rsb.info.nih.gov/ij/).

### 2.5. Statistical Analysis

GraphPad Prism (version 5.00, GraphPad Software, La Jolla, CA) was used to analyze all data. For all statistical analyses used, the alpha level was set at *p* < 0.05. Self-administration and extinction learning data were analyzed with mixed-factorial 2-way analyses of variance (ANOVA) with time as the repeated measure (2-way RM ANOVA). Group differences in lever pressing during the relapse test were investigated using one-way ANOVA (Experiment 1) or two-way ANOVA (Experiment 2). Immunoblotting data, represented by integrated density of individual protein bands, were normalized for the density of calnexin or syntaxin-1a immunoreactivity within the same sample and the treatment groups were compared using two-way ANOVAs. Significant main effects and/or interactions were followed by Bonferroni post hoc analyses to examine group or time differences.

## 3. Results

**Figure 1:**
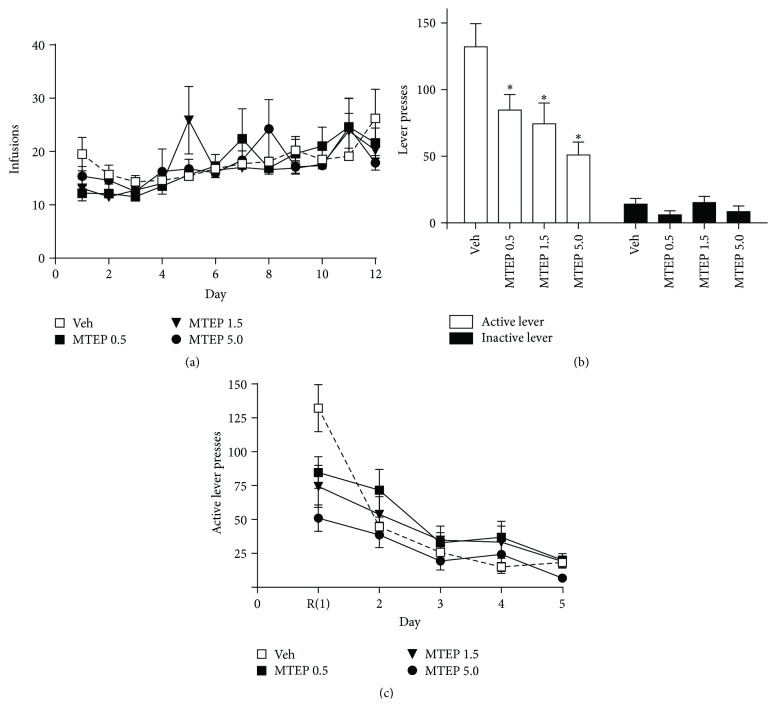
Systemic administration of MTEP attenuates context-primed relapse following abstinence and extinction learning. (a) Mean infusions during the self-administration period did not differ between groups later given MTEP or vehicle during the relapse test. (b) MTEP (0.5–5 mg/kg i.p.) significantly decreased responding on the previously active lever while not affecting inactive lever pressing. (c) Administration of MTEP prior to relapse test (R, extinction day 1) did not significantly alter extinction responding on the following days (no group differences on days 2–5). ^*∗*^ *p* < 0.05 compared to Veh.

*Experiment 1*. Mean cocaine infusions did not differ between groups later assigned to receive MTEP or vehicle, as a 2-way RM ANOVA found no significant effect of group (Figure 1(a)). While a group × time interaction was detected (*F*(33,352) = 1.606, *p* < 0.05), post hoc tests did not reveal significant differences in cocaine intake between groups. A significant effect of time was found (*F*(11,352) = 7.095, *p* < 0.001) as animals increased cocaine intake over the course of the experiment. When examining active lever presses during self-administration we found no effects of group, time, and no interaction (data not shown). We found no effects of group nor a group × time interaction when looking at inactive lever presses. However, a significant effect of time was present (*F*(11,352) = 4.994), as animals decreased left lever pressing over time (data not shown). A significant effect of group was found on active lever pressing during the relapse test (*F*(3,32) = 5.891, *p* < 0.01), and post hoc analyses revealed that rats treated with all doses of MTEP displayed significantly less presses on the previously active lever during this test relative to Veh-treated rats (*p* < 0.05; Figure 1(b)). No significant effect of group on inactive lever presses during the relapse test was detected. There was no group effect on extinction lever pressing (Figure 1(c)); however, a significant effect of time was detected (*F*(11,128) = 48.54, *p* < 0.001) due to the decrease in lever pressing over the course of extinction training. A significant group × time interaction was detected for the extinction data (*F*(3,32) = 4.596, *p* < 0.001), indicating that the groups differed in their responding over time. This effect was likely driven by the day 1 data as post hoc tests revealed that groups did not differ from each other on subsequent extinction days.

**Figure 2:**
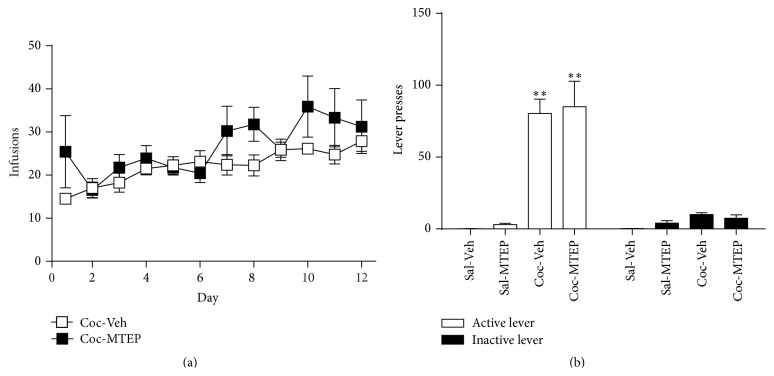
Intra-dlSTR infusion of MTEP does not attenuate context-primed relapse. (a) Mean infusions during the self-administration period did not differ between groups later given MTEP (5 *μ*g) or vehicle during the relapse test. (b) Animals that self-administered cocaine relapsed upon placement in the self-administration context following abstinence while those receiving yoked-saline infusions did not. MTEP did not alter relapse. ^*∗∗*^ *p* < 0.001 compared to Sal.

*Experiment 2*. Mean cocaine infusions did not differ between groups later assigned to receive MTEP or vehicle, as a 2-way RM ANOVA found no significant effect of MTEP and no MTEP × time interaction (Figure 2(a)). A significant effect of time was found (*F*(11,165) = 3.561, *p* < 0.001) as animals increased cocaine intake over the course of the experiment. No effects of MTEP, time, or MTEP × time interactions were detected when examining both active and inactive lever pressing during self-administration (data not shown). A two-way ANOVA was conducted on the active lever presses during the relapse test and revealed a significant effect of cocaine [Figure 2(b), *F*(1,26) = 43.32, *p* < 0.001] but no effect of MTEP and no interaction. Thus, only animals that self-administered cocaine (and not saline) relapsed and MTEP had no effect on this behavior. The same analysis was conducted on inactive lever presses during the test and found no effect of cocaine, MTEP, and no interaction (Figure 2(b)).

**Figure 3:**
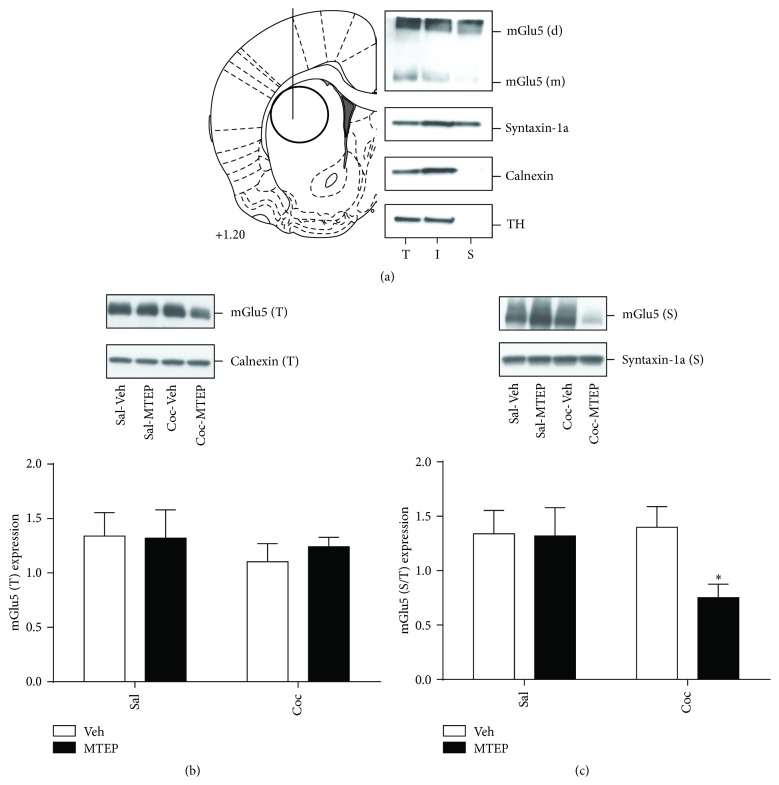
Surface expression of mGlu5 receptors in the dlSTR is reduced exclusively in animals with history of cocaine self-administration and context-primed relapse that also received intra-dlSTR infusion of MTEP. (a) Left panel: an outline of the coronal rat brain section according to Paxinos and Watson [13] demonstrating the site of drug microinjection relative to dSTR tissue dissection. Right panel: representative immunoblot analysis of fractions (T: total lysate, I: intracellular fraction, and S: surface fraction) prepared by surface biotinylation of the dlSTR slices. In addition to mGlu5, distribution of plasma membrane protein (syntaxin-1a), endoplasmic reticular protein (calnexin), and intracellular enzyme (tyrosine hydroxylase, TH) was used to monitor the cross-contamination of the prepared fractions. (b) The total protein expression of mGlu5 is not altered by cocaine or MTEP. (c) Surface protein expression of mGlu5 is reduced in animals that self-administered cocaine and received intra-dlSTR MTEP prior to the relapse test. ^*∗*^ *p* < 0.05 compared to Sal-Veh.

Animals were sacrificed 22 hrs following the end of the relapse test (24 hr following MTEP infusion) and surface biotinylation technique was employed to label and separate cell surface proteins present in the total protein lysate obtained from dlSTR slices. A representative result of a surface biotinylation experiment in dlSTR is displayed in Figure 3(a). A two-way ANOVA was used to examine effects of cocaine/saline and MTEP/Veh treatment on (surface and total) protein expression of mGlu5 and AMPA receptors as well as on total protein expression of their scaffolding/signaling partners. No effect of either variable was found on total and surface mGlu5 expression (Figures 3(b) and 3(c)). However, as we had hypothesized that the deficit in extinction learning during the four days following a single intra-dlSTR infusion of MTEP* in cocaine animals* [7] was due to alterations in mGlu5 surface expression on days following the infusion, we conducted a *t*-test on the surface mGlu5 expression data to compare only the two groups that had self-administered cocaine. We found that, in comparison to Coc-Veh animals, Coc-MTEP animals showed significantly reduced mGlu5 surface expression [Figure 3(c); *t*(1,13) = 2.752, *p* < 0.05].

Next, analysis of several proteins known to regulate mGlu5 surface trafficking was conducted in total protein lysate preparations obtained from dlSTR as described above. The proteins analyzed (CAMKII, Dynamin II/III, GRK2, Homer 1b/c, and Homer 2a/b) were selected on the basis that they participate in a cellular mechanism/pathway regulating mGlu5 surface expression. GRK2 was found to be significantly increased in rats with a history of cocaine relative to saline [Table 1; *F*(1,23) = 5.718, *p* < 0.05]; however no effect of MTEP was observed. Neither history of cocaine self-administration nor MTEP infusion altered the expression of the other proteins studied (see Table 1).

**Figure 4:**
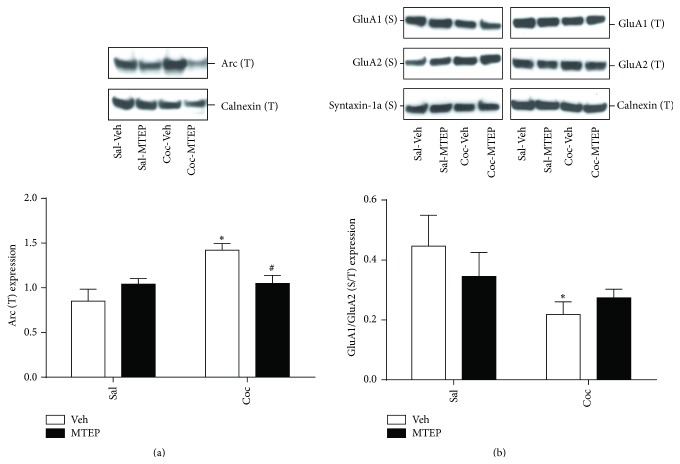
The expression of Arc and the ratio of GluA1/GluA2 surface expression are altered by cocaine history and intra-dlSTR administration of MTEP. (a) Arc expression was significantly increased by cocaine and this effect was prevented in rats that were treated with intra-dlSTR MTEP prior to the relapse test. (b) The ratio of GluA1/GluA2 was reduced significantly in Veh-treated cocaine rats relative to Veh-treated Sal control rats. ^*∗*^ *p* < 0.05 compared to Sal-Veh. ^#^ *p* < 0.5 compared to Veh-Coc.

Inhibition of mGlu5 activity produces a number of cellular changes, including decreased immediate early gene expression and changes in AMPA receptor trafficking [17, 18].  Figure 4 depicts the effects of intra-dlSTR MTEP infusion on the expression of the immediate early gene Arc and on surface expression of AMPA receptor subunits 22 hrs after the end of the relapse test. A two-way ANOVA conducted on Arc expression revealed a significant effect of cocaine [Figure 4(a); *F*(1,23) = 8.117, *p* < 0.01] and a significant cocaine × MTEP interaction, [*F*(1,23) = 7.620, *p* < 0.01]. Post hoc tests revealed that Coc-Veh rats displayed greater Arc immunoreactivity than Sal-Veh (*p* < 0.01) and MTEP reversed this effect, decreasing Arc in MTEP-treated cocaine animals relative to Coc-Veh rats (*p* < 0.05). There was no effect of either variable on surface GluA1 or GluA2 expression relative to Sal-Veh rats (data not shown). However, computing a ratio of GluA1/GluA2 reveals a significant effect of cocaine on GluA1/GluA2 expression [Figure 4(b); *F*(1,23) = 5.74, *p* < 0.05], with no effect of MTEP and no significant MTEP × cocaine interaction. Post hoc tests revealed Coc-decreased GluA1/GluA2 immunoreactivity in dlSTR in Coc-Veh, when compared to Sal-Veh animals (*p* < 0.05).

## 4. Discussion

We found that context-primed relapse to cocaine-seeking after abstinence was attenuated by acute systemic negative allosteric modulation of mGlu5 receptors (Figure 1(b)). Previously, systemic administration of MTEP has been demonstrated to attenuate reinstatement following extinction training [19, 20]. Our results are the first to provide evidence that systemic administration of MTEP attenuates context-primed relapse following abstinence. However, unlike when MTEP is infused directly into the dSTR [7], we saw no lasting effects on extinction learning after systemic administration of MTEP.

We have previously demonstrated that MTEP infused into the NAc attenuates context-induced relapse without affecting subsequent extinction learning, but MTEP infused into the dlSTR produced long-lasting effects on extinction learning after just one MTEP infusion [7]. Thus, the attenuation of relapse here is likely due to the actions of MTEP in the NAc (and potentially other brain regions). As we did not observe extinction learning deficits here, the effects of MTEP in the dlSTR have been negated upon systemic administration. This discrepancy could also be a result of different extracellular concentrations of MTEP reaching the dlSTR following systemic versus local administration [21]. Negative allosteric modulators (NAMs) of mGlu5 such as MTEP have consistently demonstrated efficacy at reducing drug-seeking and represent a valid treatment strategy for human addicts. However, their potential for negatively impacting learning is a concern. Antagonism of mGlu5 impairs performance on spatial and working memory tasks, and mGlu5 knockout mice demonstrate impaired extinction of both conditioned fear [22–24] and conditioned cocaine cues [25]. When animals enter extinction training immediately following cocaine self-administration, MTEP given daily after extinction sessions increases the number of sessions required to meet extinction criteria [26]. Conversely, daily administration of the mGlu5 positive allosteric modulator CDPPB facilitates extinction of a cocaine contextual memory and of the instrumental response made to earn cocaine [21, 27]. In summary, while chronic administration of positive and negative modulators of mGlu5 enhances and impairs extinction learning, respectively, a single systemic injection here did not have long-lasting effects on extinction learning on subsequent days.

Next, in order to examine the underlying causes of the extinction deficit that is present for four days after intra-dlSTR MTEP, we treated animals with intra-dlSTR MTEP in the same manner as we did previously [7] and sacrificed animals at the time at which they would have entered the operant box on day 2 of extinction training (i.e., 22 hr after relapse test; 24 hr after Veh or MTEP infusion). Surface biotinylation analysis of the dlSTR revealed decreased surface expression of mGlu5 in animals that self-administered cocaine and received MTEP infusion relative to vehicle-infused cocaine rats (Figure 3(c)), potentially providing a mechanism for the long-lasting effects of intra-dlSTR MTEP on extinction learning observed previously [7]. Vehicle-treated cocaine animals displayed equal mGlu5 levels as vehicle-treated yoked-saline controls (Figure 3(c)), in contrast to our previous finding of a significant reduction in surface mGlu5 in cocaine animals relative to yoked-saline controls when animals were killed* prior* to the relapse test [7]. As the decrease of mGlu5 in cocaine animals precedes intra-dlSTR infusion and the relapse test, we hypothesize that (1) cocaine-seeking increased mGlu5 surface expression in Coc-Veh animals effectively “normalizing” mGlu5 surface levels, and (2) this increase was prevented by local administration of MTEP. It should be noted that total mGlu5 levels were not altered in all experimental conditions, suggesting changes in internalization/surface delivery rather than degradation of receptors. In support of this, recent evidence suggests that internalized mGlu5 receptors typically undergo recycling (membrane reinsertion), not degradation [28].

In an attempt to investigate possible mechanisms facilitating mGlu5 surface delivery exclusively in Coc-Veh animals, as well as the inhibitory mechanisms of MTEP preventing this delivery, levels of several mGlu5-interacting proteins were measured across all treatment groups. Candidate proteins were selected based on their (1) previously described interaction with mGlu5, (2) known effects on mGlu5 surface trafficking, and (3) moderate-to-high expression levels in the dlSTR. Thus, we chose to quantify Homer 1b/c and Homer 2a/b [29, 30], Dynamin II/III [31, 32], GRK2 [33], and CAMKII [34, 35] levels. Surprisingly, with the exception of increased GRK2 levels, no other changes in total protein expression were detected (Table 1). Ribeiro et al. [33] observed that upregulation of GRK2 levels in the striatum promotes agonist-independent internalization of mGlu5. However, GRK2-mediated mGlu5 internalization cannot explain different mGlu5 surface levels in Coc-Veh versus Coc-MTEP animals as GRK2 was upregulated in both groups. Therefore we can hypothesize that the GRK2 increase is related to a loss of mGlu5 surface expression detected in all cocaine animals prior to the relapse test [7] and has no relationship to the “normalization” of mGlu5 surface levels 22 hr after the relapse in the Coc-Veh group only. Further, we hypothesize that restoration (normalization) of mGlu5 function in Coc-Veh represents a homeostatic mechanism facilitating extinction learning, and inability to “normalize” mGlu5 surface expression in Coc-MTEP animals is related to impairment of extinction learning observed in our previous study [7]. This hypothesis will be tested in the future studies.

While the role of dlSTR glutamate and mGlu5 receptors in regulating drug-seeking depends on the presence of discrete cues during the self-administration training as well as on the extent of training [10, 36, 37], activity of mGlu5 receptors in the striatum is necessary for extinction learning [7, 38]. Therefore, in the current study we evaluated two well-characterized downstream events: the expression of an effector immediate early gene Arc and surface trafficking of AMPA receptors, both measured at the time point rats would have entered the operant chambers for extinction day 2. Administration of mGlu5 agonists or reexposure of animals to a drug-paired context induces Arc expression in the striatum [39–42]. While most studies describe a rapid, short-lived pattern of Arc expression following the stimulus, in some behavioral paradigms involving conditioned learning a biphasic pattern of Arc expression has been observed [43, 44]. This corresponds to our finding of increased Arc protein levels in the dlSTR 22 hr following the reexposure to drug-paired context (relapse test) in the Coc-Veh group. As Arc is considered critical for activity-dependent synaptic plasticity, learning, and memory [for review see [45]], the delayed rise in Arc expression might be related to its role in memory (re)consolidation important for extinction learning. The upregulation of Arc expression observed here in Coc-Veh but not Coc-MTEP rats (Figure 4(a)) may explain our previous report that Coc-Veh animals display accelerated extinction learning when compared to Coc-MTEP animals [7]. In support of this idea, we have previously found that knocking down Arc levels in the dlSTR impairs extinction of responding following context-induced relapse [39].

One of the well-documented consequences of Arc upregulation is internalization of AMPA receptors [17, 18, 46]. At least in the hippocampus, Arc-mediated internalization of AMPA receptors is contingent upon activation of mGlu5 receptors [18]. Depending on experimental conditions, Arc either did not discriminate between GluA1- and GluA2-containing AMPA receptors [46, 47] or selectively internalized GluA1-containing receptors [17, 48]. In this latter scenario, Arc would mediate a selective removal of GluA1-containing receptors, decreasing the ratio of GluA1-to-GluA2 on the cell surface. Indeed, this is in agreement with current findings in Coc-Veh (but not Coc-MTEP) animals 22 hr after the relapse test (Figure 4(b)). As this time point corresponds to impaired extinction learning in Coc-MTEP animals [7], we can hypothesize that MTEP prevented GluA1/GluA2 modification that is necessary for weakening of synaptic plasticity and extinction learning [18, 49]. In support of this idea, it has been shown that extended extinction training alters GluA1, but not GluA2 protein levels following cocaine-cue extinction learning [50, 51].

## 5. Conclusions

The data presented here indicate that systemic treatment with MTEP attenuates relapse of cocaine-seeking induced by the drug self-administration context and not maintained by the presentation of conditioned cues. Thus, medications that block mGlu5 are viable treatments for cocaine relapse, as systemic administration of these medications also attenuates cue- and cocaine-primed reinstatement following extinction training [14, 15]. We also found decreased mGlu5 surface expression in the dlSTR of cocaine-self-administering rats that received local infusion of MTEP, but not Veh. Based on our previous findings of decreased dlSTR mGlu5 surface expression prior to relapse in cocaine-self-administering rats relative to cocaine-naïve rats [7], we hypothesize that the normalization of mGlu5 surface expression that coincides with Arc induction and the decreased surface ratio of GluA1/GluA2 containing AMPA receptors is necessary for postabstinence extinction learning in cocaine animals and this signaling cascade is impaired by local MTEP infusion. Therefore, the undesirable effects of intra-dSTR MTEP administration on extinction learning are likely occurring because MTEP prevents the restoration of mGlu5 surface expression that occurs as a result of drug-seeking and/or extinction. In conclusion, while the use of mGlu5 NAMs might diminish relapse risk in drug addiction, potential learning and memory impairments stemming from higher doses or prolonged administration of mGlu5-targeting drugs require further preclinical evaluation.

## Figures and Tables

**Table 1:** Immunoreactivity of mGlu5-interacting proteins in the dlSTR 22 hr after the context-induced relapse test.

Protein	Treatment group			
	Sal-Veh	Sal-MTEP	Coc-Veh	Coc-MTEP
CAMK II	0.724 ± 0.093	0.883 ± 0.058	0.840 ± 0.095	0.723 ± 0.067
Dynamin II/III	0.815 ± 0.095	0.830 ± 0.101	0.918 ± 0.068	0.780 ± 0.071
GRK2	0.553 ± 0.046	0.460 ± 0.068	0.629 ± 0.068^*∗*^	0.702 ± 0.058^*∗*^
Homer 1b/c	0.404 ± 0.044	0.413 ± 0.050	0.450 ± 0.051	0.374 ± 0.041
Homer 2a/b	1.347 ± 0.141	1.500 ± 0.117	1.562 ± 0.184	1.372 ± 0.144

All data were normalized to calnexin control (within sample) and to the Sal-Veh control (between groups). Data represent mean immunoreactivity ± SEM (*n* = 5–9). ^*∗*^ *p* < 0.05 compared to Sal.

## References

[1] O'Brien C., Hardman J. L. L., Gilman A. G. (2001). Drug addiction and drug abuse. *The Pharmacological Basis of Therapeutics*.

[2] Mendelson J. H., Mello N. K. (1996). Management of cocaine abuse and dependence. *The New England Journal of Medicine*.

[3] Shaham Y., Stewart J. (1995). Stress reinstates heroin-seeking in drug-free animals: an effect mimicking heroin, not withdrawal. *Psychopharmacology*.

[4] Meil W. M., See R. E. (1996). Conditioned cued recovery of responding following prolonged withdrawal from self-administered cocaine in rats: an animal model of relapse. *Behavioural Pharmacology*.

[5] de Wit H., Stewart J. (1981). Reinstatement of cocaine-reinforced responding in the rat. *Psychopharmacology*.

[6] Rothstein J. D., Martin L., Levey A. I. (1994). Localization of neuronal and glial glutamate transporters. *Neuron*.

[7] Knackstedt L. A., Trantham-Davidson H. L., Schwendt M. (2014). The role of ventral and dorsal striatum mGluR5 in relapse to cocaine-seeking and extinction learning. *Addiction Biology*.

[8] Miller S., Kesslak J. P., Romano C., Cotman C. W. (1995). Roles of metabotropic glutamate receptors in brain plasticity and pathology. *Annals of the New York Academy of Sciences*.

[9] McLaughlin J., See R. E. (2003). Selective inactivation of the dorsomedial prefrontal cortex and the basolateral amygdala attenuates conditioned-cued reinstatement of extinguished cocaine-seeking behavior in rats. *Psychopharmacology*.

[10] See R. E., Elliott J. C., Feltenstein M. W. (2007). The role of dorsal vs ventral striatal pathways in cocaine-seeking behavior after prolonged abstinence in rats. *Psychopharmacology*.

[11] Diamond J. S., Jahr C. E. (2000). Synaptically released glutamate does not overwhelm transporters on hippocampal astrocytes during high-frequency stimulation. *Journal of Neurophysiology*.

[12] Knackstedt L. A., Kalivas P. W. (2009). Glutamate and reinstatement. *Current Opinion in Pharmacology*.

[13] Paxinos G., Watson C. (2007). *The Rat Brain in Sterotaxic Coordinates*.

[14] Kumaresan V., Yuan M., Yee J. (2009). Metabotropic glutamate receptor 5 (mGluR5) antagonists attenuate cocaine priming- and cue-induced reinstatement of cocaine seeking. *Behavioural Brain Research*.

[15] Martin-Fardon R., Baptista M. A. S., Dayas C. V., Weiss F. (2009). Dissociation of the effects of MTEP [3-[(2-methyl-1,3-thiazol-4-yl)ethynyl]piperidine] on conditioned reinstatement and reinforcement: comparison between cocaine and a conventional reinforcer. *The Journal of Pharmacology and Experimental Therapeutics*.

[16] Sinclair C. M., Cleva R. M., Hood L. E., Olive M. F., Gass J. T. (2012). mGluR5 receptors in the basolateral amygdala and nucleus accumbens regulate cue-induced reinstatement of ethanol-seeking behavior. *Pharmacology Biochemistry and Behavior*.

[17] Chowdhury S., Shepherd J. D., Okuno H. (2006). Arc/Arg3.1 interacts with the endocytic machinery to regulate AMPA receptor trafficking. *Neuron*.

[18] Waung M. W., Pfeiffer B. E., Nosyreva E. D., Ronesi J. A., Huber K. M. (2008). Rapid translation of Arc/Arg3.1 selectively mediates mGluR-dependent LTD through persistent increases in AMPAR endocytosis rate. *Neuron*.

[19] Broderick P. A. (1991). Cocaine: on-line analysis of an accumbens amine neural basis for psychomotor behavior. *Pharmacology, Biochemistry and Behavior*.

[20] Garrett B. E., Holtzman S. G. (1994). D_1_ and D_2_ dopamine receptor antagonists block caffeine-induced stimulation of locomotor activity in rats. *Pharmacology, Biochemistry and Behavior*.

[21] Pulvirenti L., Swerdlow N. R., Koob G. F. (1989). Microinjection of a glutamate antagonist into the nucleus accumbens reduces psychostimulant locomotion in rats. *Neuroscience Letters*.

[22] Witkin J. M. (1993). Blockade of the locomotor stimulant effects of cocaine and methamphetamine by glutamate antagonists. *Life Sciences*.

[23] Cabib S., Castellano C., Cestari V., Filibeck U., Puglisi-Allegra S. (1991). D1 and D2 receptor antagonists differently affect cocaine-induced locomotor hyperactivity in the mouse. *Psychopharmacology*.

[24] Dalia A., Uretsky N. J., Wallace L. J. (1998). Dopaminergic agonists administered into the nucleus accumbens: Effects on extracellular glutamate and on locomotor activity. *Brain Research*.

[25] Bird M. K., Lohmann P., West B. (2014). The mGlu5 receptor regulates extinction of cocaine-driven behaviours. *Drug and Alcohol Dependence*.

[26] Kim J. H., Perry C., Luikinga S., Zbukvic I., Brown R. M., Lawrence A. J. (2015). Extinction of a cocaine-taking context that protects against drug-primed reinstatement is dependent on the metabotropic glutamate 5 receptor. *Addiction Biology*.

[27] Pulvirenti L., Swerdlow N. R., Koob G. F. (1991). Nucleus accumbens NMDA antagonist decreases locomotor activity produced by cocaine, heroin or accumbens dopamine, but not caffeine. *Pharmacology, Biochemistry and Behavior*.

[28] Trivedi R. R., Bhattacharyya S. (2012). Constitutive internalization and recycling of metabotropic glutamate receptor 5 (mGluR5). *Biochemical and Biophysical Research Communications*.

[29] Ango F., Robbe D., Tu J. C. (2002). Homer-dependent cell surface expression of metabotropic glutamate receptor type 5 in neurons. *Molecular and Cellular Neuroscience*.

[30] Shiraishi-Yamaguchi Y., Furuichi T. (2007). The Homer family proteins. *Genome Biology*.

[31] Gray N. W., Fourgeaud L., Huang B. (2003). Dynamin 3 is a component of the postsynapse, where it interacts with mGluR5 and Homer. *Current Biology*.

[32] Fourgeaud L., Bessis A.-S., Rossignol F., Pin J.-P., Olivo-Marin J.-C., Hémar A. (2003). The metabotropic glutamate receptor mGluR5 is endocytosed by a clathrin-independent pathway. *Journal of Biological Chemistry*.

[33] Ribeiro F. M., Ferreira L. T., Paquet M. (2009). Phosphorylation-independent regulation of metabotropic glutamate receptor 5 desensitization and internalization by G protein-coupled receptor kinase 2 in neurons. *The Journal of Biological Chemistry*.

[34] Jin D.-Z., Guo M.-L., Xue B., Mao L.-M., Wang J. Q. (2013). Differential regulation of CaMKII*α* interactions with mGluR5 and NMDA receptors by Ca^2+^ in neurons. *Journal of Neurochemistry*.

[35] Raka F., Di Sebastiano A. R., Kulhawy S. C. (2015). Ca^2+^/Calmodulin-dependent protein kinase II interacts with group I metabotropic glutamate and facilitates receptor endocytosis and ERK1/2 signaling: role of *β*-amyloid. *Molecular Brain*.

[36] Vanderschuren L. J. M. J., Di Ciano P., Everitt B. J. (2005). Involvement of the dorsal striatum in cue-controlled cocaine seeking. *Journal of Neuroscience*.

[37] Gabriele A., Pacchioni A. M., See R. E. (2012). Dopamine and glutamate release in the dorsolateral caudate putamen following withdrawal from cocaine self-administration in rats. *Pharmacology Biochemistry and Behavior*.

[38] Cleva R. M., Hicks M. P., Gass J. T. (2011). mGluR5 positive allosteric modulation enhances extinction learning following cocaine self-administration. *Behavioral Neuroscience*.

[39] Hearing M. C., Schwendt M., McGinty J. F. (2011). Suppression of activity-regulated cytoskeleton-associated gene expression in the dorsal striatum attenuates extinction of cocaine-seeking. *The International Journal of Neuropsychopharmacology*.

[40] Hearing M. C., See R. E., McGinty J. F. (2008). Relapse to cocaine-seeking increases activity-regulated gene expression differentially in the striatum and cerebral cortex of rats following short or long periods of abstinence. *Brain Structure and Function*.

[41] Kumar V., Fahey P. G., Jong Y.-J. I., Ramanan N., O'Malley K. L. (2012). Activation of intracellular metabotropic glutamate receptor 5 in striatal neurons leads to up-regulation of genes associated with sustained synaptic transmission including Arc/Arg3.1 protein. *The Journal of Biological Chemistry*.

[42] Schwendt M., Sigmon S. A., McGinty J. F. (2012). RGS4 overexpression in the rat dorsal striatum modulates mGluR5- and amphetamine-mediated behavior and signaling. *Psychopharmacology*.

[43] Ramírez-Amaya V., Vazdarjanova A., Mikhael D., Rosi S., Worley P. F., Barnes C. A. (2005). Spatial exploration-induced Arc mRNA and protein expression: evidence for selective, network-specific reactivation. *The Journal of Neuroscience*.

[44] Lonergan M. E., Gafford G. M., Jarome T. J., Helmstetter F. J. (2010). Time-dependent expression of arc and Zif268 after acquisition of fear conditioning. *Neural Plasticity*.

[45] Bramham C. R., Worley P. F., Moore M. J., Guzowski J. F. (2008). The immediate early gene Arc/Arg3.1: regulation, mechanisms, and function. *Journal of Neuroscience*.

[46] Rial Verde E. M., Lee-Osbourne J., Worley P., Malinow R., Cline H. (2006). Increased expression of the immediate-early gene arc/arg3.1 reduces AMPA receptor-mediated synaptic transmission. *Neuron*.

[47] Shepherd J. D., Rumbaugh G., Wu J. (2006). Arc/Arg3.1 mediates homeostatic synaptic scaling of AMPA receptors. *Neuron*.

[48] Peebles C. L., Yoo J., Thwin M. T., Palop J. J., Noebels J. L., Finkbeiner S. (2010). Arc regulates spine morphology and maintains network stability in vivo. *Proceedings of the National Academy of Sciences of the United States of America*.

[49] Park S., Park J. M., Kim S. (2008). Elongation factor 2 and fragile X mental retardation protein control the dynamic translation of Arc/Arg3.1 essential for mGluR-LTD. *Neuron*.

[50] Nic Dhonnchadha B. A., Lin A., Leite-Morris K. A., Kaplan G. B., Man H. Y., Kantak K. M. (2013). Alterations in expression and phosphorylation of GluA1 receptors following cocaine-cue extinction learning. *Behavioural Brain Research*.

[51] Zavala A. R., Biswas S., Harlan R. E., Neisewander J. L. (2007). Fos and glutamate AMPA receptor subunit coexpression associated with cue-elicited cocaine-seeking behavior in abstinent rats. *Neuroscience*.

